# Effects of long-term cultivation of transgenic *Bt* rice (*Kefeng-6*) on soil microbial functioning and C cycling

**DOI:** 10.1038/s41598-017-04997-8

**Published:** 2017-07-05

**Authors:** Lee Zhaolei, Bu Naishun, Cui Jun, Chen Xueping, Xiao Manqiu, Wang Feng, Song Zhiping, Fang Changming

**Affiliations:** 1 Ministry of Education Key Laboratory for Biodiversity and Ecological Engineering, The Institution of Biodiversity Science, Fudan University, Shanghai, China; 2 School of Environmental Science, Liaoning University, Shenyang, China; 3 School of Environmental and Chemical Engineering, Shanghai University, Shanghai, China; 4 Fujian Province Key Laboratory of Genetic Engineering for Agriculture, Fujian Academy of Agricultural Sciences, Fuzhou, China

## Abstract

Understanding how soil ecosystem responds to transgenic *Bacillus thuringiensis* (*Bt*) rice is necessary for environmental risk assessment. While the influences of short-term cultivation of *Bt* rice on soil properties have been reported previously, little is known about the long-term effects of *Bt* rice on soil ecosystems. In this study, soil samples were taken from a long-term rice cultivation site in Fujian Province, China, where transgenic *Bt* rice (*Kefeng-6*) and its non-*Bt* parent breed (*Minghui-86*) had been continuously cultivated for 8 years. Soil *Bt* protein concentration and a total of 16 variables were analyzed to assess potential risks of soil health under *Bt* rice cultivation. The results revealed that soil *Bt* protein is unlikely to accumulate after *Bt* rice cultivated in the field, and no consistently significant changes were observed in soil enzymatic activities (catalase, dehydrogenase, acid phosphatase, and urease), microbial biomass (microbial carbon and nitrogen), total organic carbon, decomposition (soil respiration, *Q*_10_, and *q*CO_2_), soil nitrogen and phosphorus contents. Due to a local tradition that aboveground biomass was removed after harvest, the increased net primary productivity by *Bt* rice cultivation did not significantly change soil C cycling. Results of this study suggested that on the aspects of soil microbial functioning and C cycling, long-term cultivation of *Bt* rice is unlikely to result in significant effects on soil health.

## Introduction

Transgenic *Bacillus thuringiensis* (*Bt*) crops are genetically modified to express certain larvicidal toxins to kill target pests. These insect-resistant crops have a great advantage in agriculture by reducing pesticide application^1^. Transgenic *Bt* crops, however, release *Bt* protein into environments through above- and below-ground debris and root exudates, which may lead to profound impacts on agro-ecosystems. Previous studies have reported that transgenic *Bt* crops may beget compound effects on agro-ecosystems, such as gene flow between plant species^2^, developing more resistance to *Bt* protein in target insects^3–5^, and potential impacts on non-target insects^6–8^. Soil is the core of agro-ecosystem, supporting crop growth, serving as a nutrient reservoir and providing the habitat of belowground organisms that mediate terrestrial nutrient cyclings. It is imperative to understand whether soil health has been significantly influenced by *Bt* crops.

It was reported that *Bt* protein from *Bt* crops could persist in the soil, mainly through binding to humus or clay^9–11^, and remained functioning for a relatively long period^12^. On the aspect of soil health, it is important to understand whether *Bt* crops can significantly change soil biota, microbial activities and ecological processes (*e*.*g*., soil carbon and nutrient cyclings). A large number of previous studies have been conducted to explore possible effects of *Bt* crops on soil ecosystem but obtained inconsistent results. Li and Liu^13^ found that the richness and diversity of nematode were not significantly altered by *Bt* cotton. Similar results were reported on herbivory nematode^14^, earthworm and protozoa^15^. Besides, a few studies pointed out that *Bt* crops did not significantly change soil microbial biomass^16^ or soil enzymatic activities^17–19^. Soil total organic carbon (TOC) and total nitrogen (TN) pools were insensitive to cultivation of *Bt* crops^20–22^. *In situ* soil respiration rates did not significantly differ between *Bt* and non-*Bt* crops^18, 23^. On the other hand, it was reported that growing *Bt* crops might cause some significant changes in soil variables related to soil health. For instance, *Bt* cotton could increase the diversity of fungi and bacteria^24^. *Bt* crops enhanced dehydrogenase^17, 25^ and urease activities^26^. Fliessbach *et al*.^25^ found that *Bt* maize increased the dehydrogenase activity by 6%. In addition, soil respiration could be significantly increased^27, 28^ or decreased^25^ by transgenic *Bt* maize in different studies. Most of these reported studies were based on short-term field experiments, up to two or three years^13, 29, 30^, enabling us to learn what and how *Bt* crops affect soil microbial organisms and soil eco-processes. However, short-term cultivation experiments encountered temporal variations in soil environment and crop growth, were not able to reflect the accumulative effects of *Bt* protein along with the food web in the soil, it is still poorly understood whether long-term cultivation of *Bt* crops will result in irreversible impacts on soil health.

Furthermore, previous studies almost centered on *Bt*-corn and *Bt*-cotton^18, 25, 26^ and it is indispensable to evaluate soil health under *Bt* rice cultivation according to the case-by-case principle of biosafety assessment. Rice is one of major food sources in the world, and rice planting area covers more than 150 million hectares^31^. In China, many breeds of transgenic *Bt* rice have been reported to promote food production and to reduce pesticide applications^1^, but none has been commercially released to date. Transgenic *Bt* rice is likely to be commercially released in near future, to meet increasing food demand. It is an urgent task to understand potential threats to soil health of long-term cultivation of transgenic *Bt* rice. Under the canopy of *Bt* rice, soil microbial biomass declined by 7%^32^; dehydrogenase activity increased by 95% at the initial stage of straw decomposition but decreased by 47% at subsequent stages^33^; soil respiration rate increased by 25%^34^. All of these studies were based on short-term experiments (1–3 years), mainly due to the lack of long-term experimental platform of transgenic *Bt* rice cultivation.

Using knowledge from short-term cultivation of *Bt* rice may not reliably predict the long-term effects of *Bt* rice on soil ecosystem, because that significant shifts in soil traits might occur after lasting accumulation of slight changes caused by *Bt* rice, or that these slight changes might offset each other, resulting in no significant long-term effects on soil ecosystem. It is necessary and urgent to assess the long-term effects of *Bt* rice on soil ecosystem before *Bt* rice is commercially released and widely planted. Because of the lack of long-term experimental platform, assessing the potential environment risk of *Bt* rice is still a difficult task. In this study, we took advantage of an experimental platform for breeding *Bt* rice *Kefeng-6* (encoding *Cry1Ac* protein), the longest one of *Bt* rice cultivation in China where *Bt* rice had been continuously cultivated for 8 years when we conducted this study, to assess the possible effects of *Bt* rice on soil health. The objectives of this study were to investigate: (1) whether long-term cultivation of *Bt* rice causes the accumulation of *Bt* protein in soil; (2) whether there are irreversibly negative effects of *Bt* rice cultivation on soil properties mediating soil carbon and nitrogen cycling and maintaining ecosystem health, such as enzymatic activities, microbial biomass, and dynamics of C pool and decomposition.

## Results

### Plant biomass, C/N ratio, and *Bt* protein concentration

**Figure 1:**
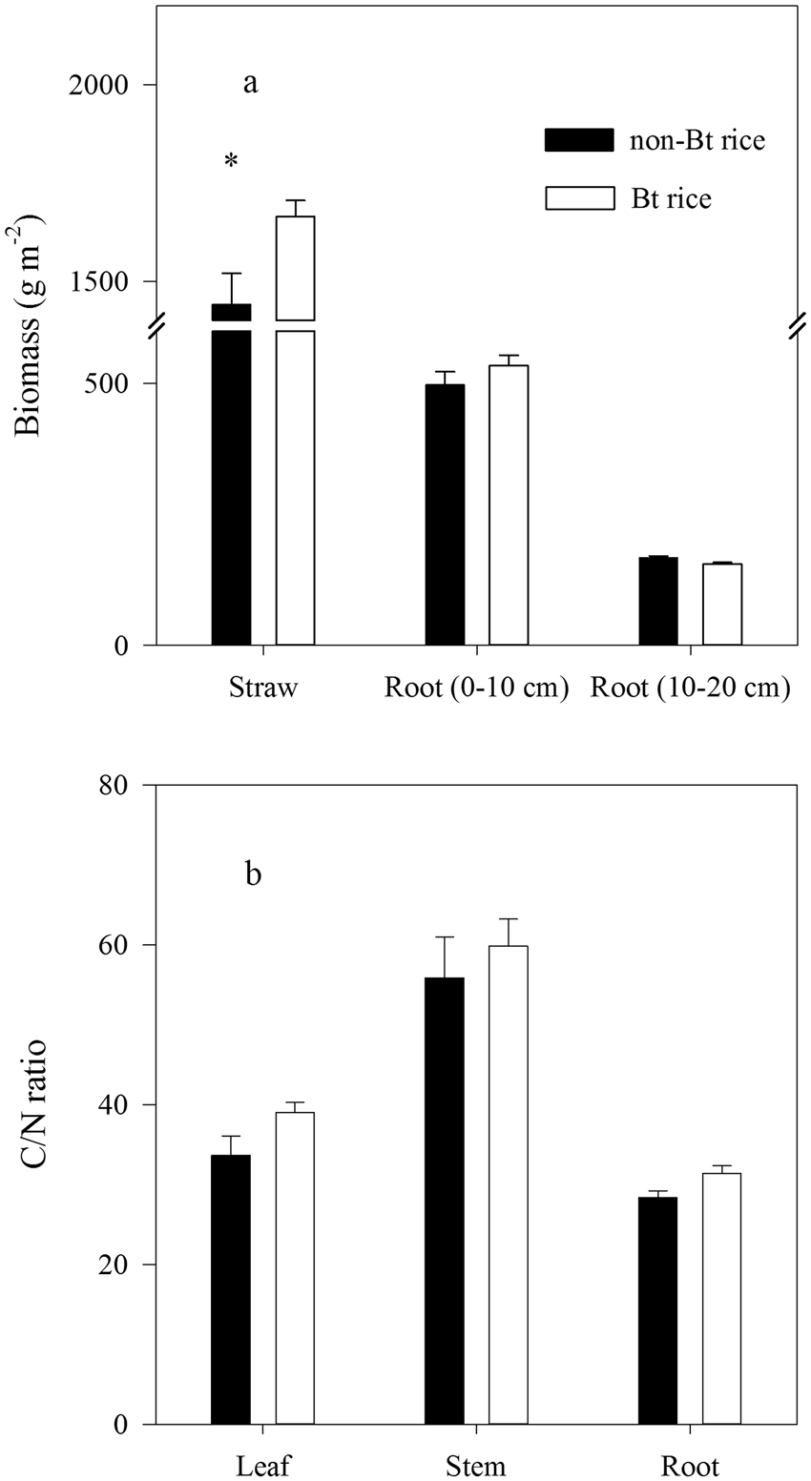
Straw and root biomass of top- (0–10 cm) and sub-soil (10–20 cm) (**a**) and C/N ratio in root, leaf and stem (**b**) of *Bt* rice treatments. Error bars indicate standard error (n = 4) and star indicates the significant difference in biomass.

The straw biomass of *Bt* rice was significantly higher than that of non-*Bt* rice (*t* = 2.85, *p* < 0.05; Fig. 1a). However, no significant differences were observed in root biomass between *Bt* and non-*Bt* rice, either in soil layer of 0–10 cm (*t* = 1.14, *p* = 0.29) or 10–20 cm (*t* = −2.03, *p* = 0.08). The C/N ratios of root, stem and leaf were similar between *Bt* rice and its parental breed (*t* = 0.87, *p* = 0.43; *t* = 0.19, *p* = 0.10; *t* = 0.68, *p* = 0.53, respectively; Fig. 1b). *Bt* protein was not detected out using enzyme-linked immunosorbent assay (ELISA) approach in soil samples collected from *Bt* treatment.

### Enzymatic activities

**Figure 2:**
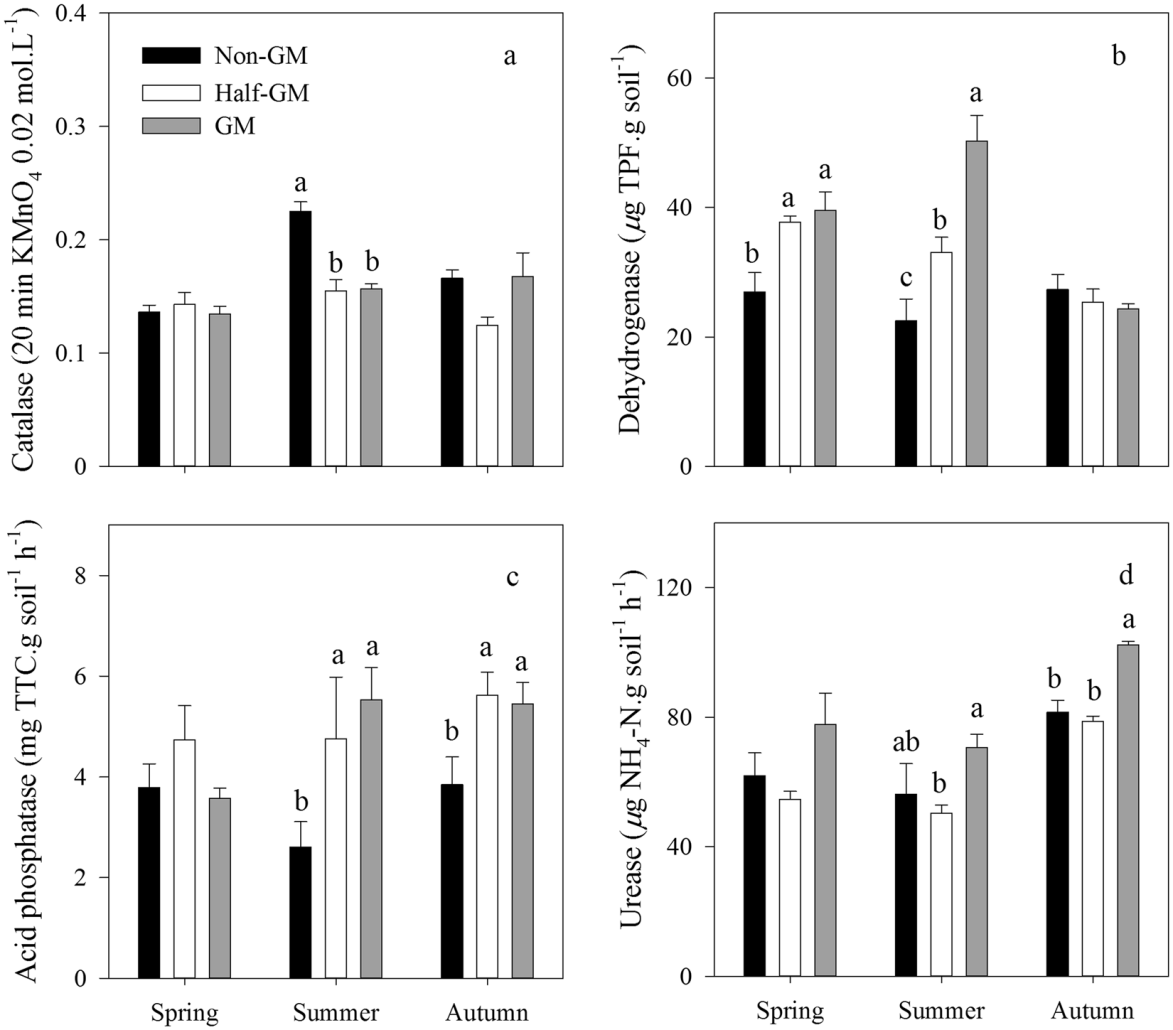
Seasonal variations in the activities of soil catalase (**a**), dehydrogenase (**b**), acid phosphatase (**c**) and urease (**d**) of *Bt* treatments. Error bars represent the standard error (n = 4). The same letters over error bars denote no significant difference between treatments (*p* > 0.05).

In general, there were no consistently significant differences in soil (0–20 cm) catalase activity among all three treatments (Fig. 2a). During summer time when rice was growing, catalase activity was significantly higher in *Non*-*GM* than those in other treatments. In autumn after rice harvest, *Half*-*GM* had a lower soil catalase activity than *GM* and *Non-GM*, but the differences were not statistically significant (*F* = 3.55, *p* = 0.07).

Differences in dehydrogenase activity among treatments varied over time (Fig. 2b). During spring and summer, dehydrogenase activities in *Non*-*GM* were significantly lower than those in *GM* and *Half*-*GM* (*F* = 7.61, *p* = 0.01; *F* = 16.56, *p* < 0.001). After rice harvest, soil samples of three treatments exhibited similar level of dehydrogenase activities (*F* = 0.66, *p* = 0.54).

In spring before rice planting, differences in acid phosphatase activity among treatments were not significant (*F* = 1.58, *p* = 0.26; Fig. 2c). During summer and autumn, *Non*-*GM* had significantly lower acid phosphatase activity than *Half*-*GM* and *GM* (*F* = 15.92, *p* < 0.001; *F* = 4.15, *p* = 0.05). No consistently significant differences in phosphatase activity were observed between *GM* and *Half*-*GM* treatments throughout the year.

Although *Half*-*GM* had the lowest urease activity among the three treatments through the year (Fig. 2d), the differences were significant only between *Half*-*GM* and *GM* treatments.

### Soil respiration

**Figure 3:**
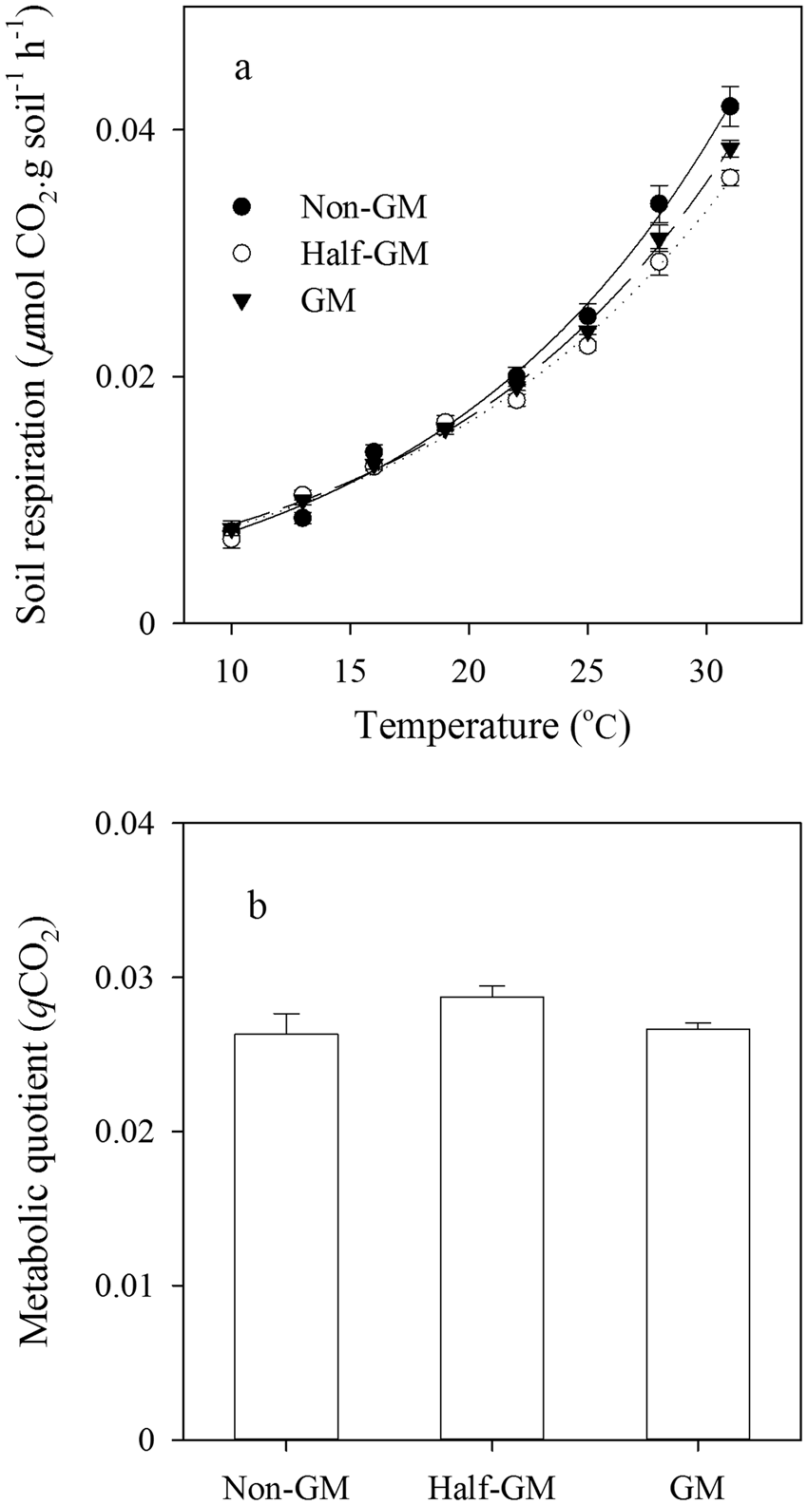
The relationship of soil respiration with incubation temperature (**a**) and microbial metabolic quotient (**b**) of different treatments. Error bars indicated standard error (n = 4).

Figure 3a shows soil respiration variation along with incubation temperature for soil samples (0–20 cm) taken in summer time. Soil respiration rate against temperature in other seasons was similar (data not shown). The reference respiration rates (*R*_20_) were 0.0168 ± 0.0003, 0.0165 ± 0.0003, and 0.0173 ± 0.0006 *μ*mol C g^−1^ dry soil h^−1^ for *GM*, *Half-GM* and *Non-GM*, respectively. Estimated *Q*_10_ values of soil respiration were 2.34 ± 0.04, 2.24 ± 0.03 and 2.38 ± 0.10 for *GM*, *Half-GM* and *Non-GM* treatment, respectively, suggesting that the temperature sensitivity of TOC decomposition was not significantly influenced by treatments (*F* = 0.94, *p* = 0.41). Similarly, soil microbial metabolic quotients (*q*CO_2_) were similar among treatments (*F* = 2.08, *p* = 0.18), indicating that the overall microbial activity was not significantly changed by different treatments (Fig. 3b).

### Soil carbon and nutrients

**Figure 4:**
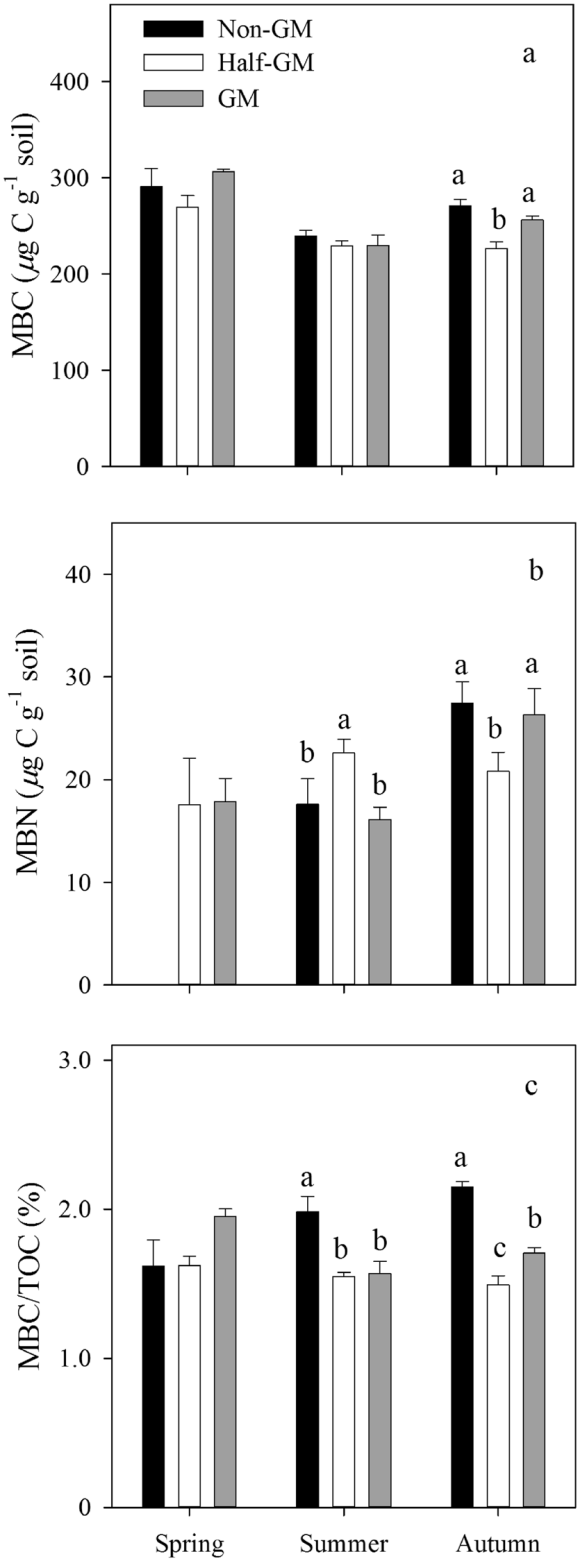
Seasonal variations in soil microbial biomass carbon (**a**), microbial biomass nitrogen (**b**) and the ratio of MBC/TOC (**d**) of *Bt* treatments. Error bars represent standard error (n = 4). The same letters over error bars denote no significant difference between treatments (*p* > 0.05).

No consistent differences in soil microbial biomass carbon (MBC) and nitrogen (MBN) were observed among *Bt* treatments along with time (Fig. 4a and b). Although MBC in *Half-GM* was the lowest, the differences were statistically significant only in autumn after rice harvest (*F* = 14.09, *p* = 0.001). MBN in *Half-GM* was the highest in summer (*F* = 5.10, *p* = 0.02), but the lowest in autumn (*F* = 14.09, *p* = 0.001). In spring before rice planting, the ratio of MBC/TOC in *GM* was higher than those in *Half*-*GM* and *Non*-*GM*. However, *Non*-*GM* had the highest ratios in summer and autumn, significantly greater than those in other two treatments (*F* = 10.48, *p* = 0.002; *F* = 56.34, *p* < 0.001; Fig. 4c).

**Figure 5:**
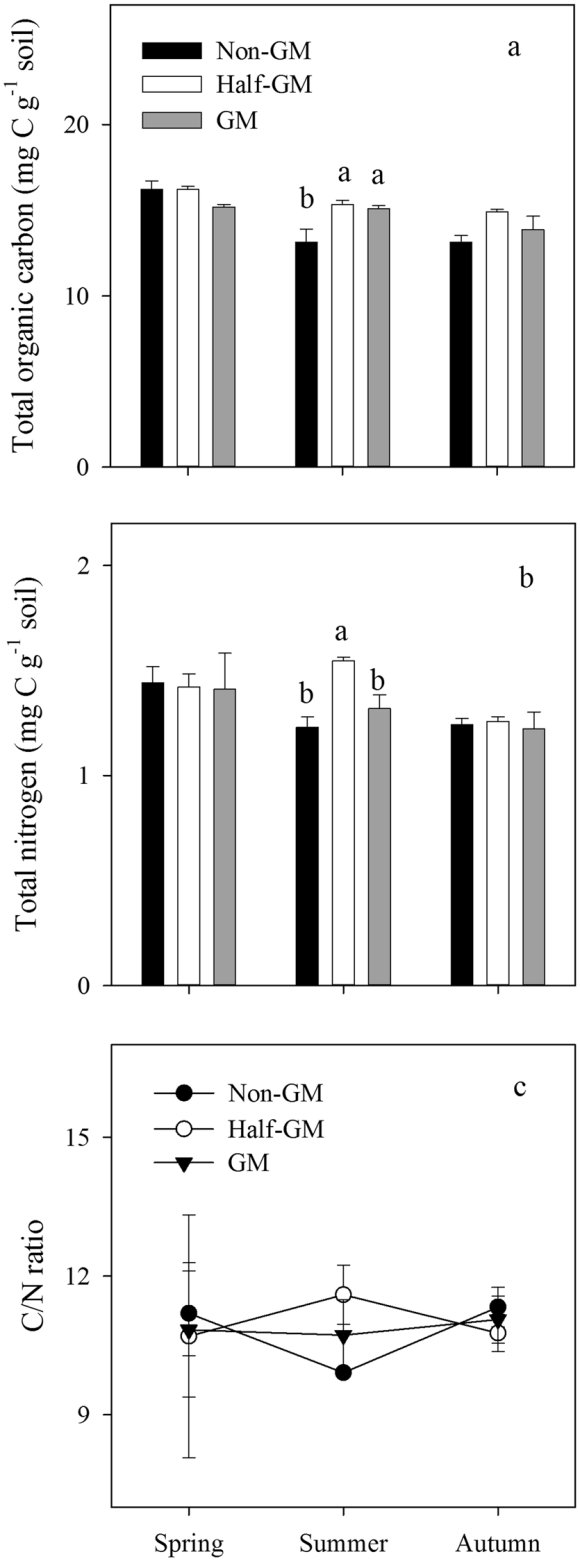
Seasonal variations of total organic carbon (**a**), total nitrogen (**b**) and C/N ratio (**c**) in the soil of *Bt* treatments. Error bars represent standard error (n = 4). The same letters over error bars denote no significant difference between treatments (*p* > 0.05).

TOC and TN were at similar levels among the three treatments, except in summer when TOC and TN were significantly higher in *Half*-*GM* than those in *Non-GM* (*F* = 8.26, *p* = 0.005). The annual mean C/N ratios were 10.87, 11.01 and 10.81 for *GM*, *Half-GM* and *Non-GM*, respectively, suggesting no systematically significant effect by *Bt* treatments (Fig. 5).

**Figure 6:**
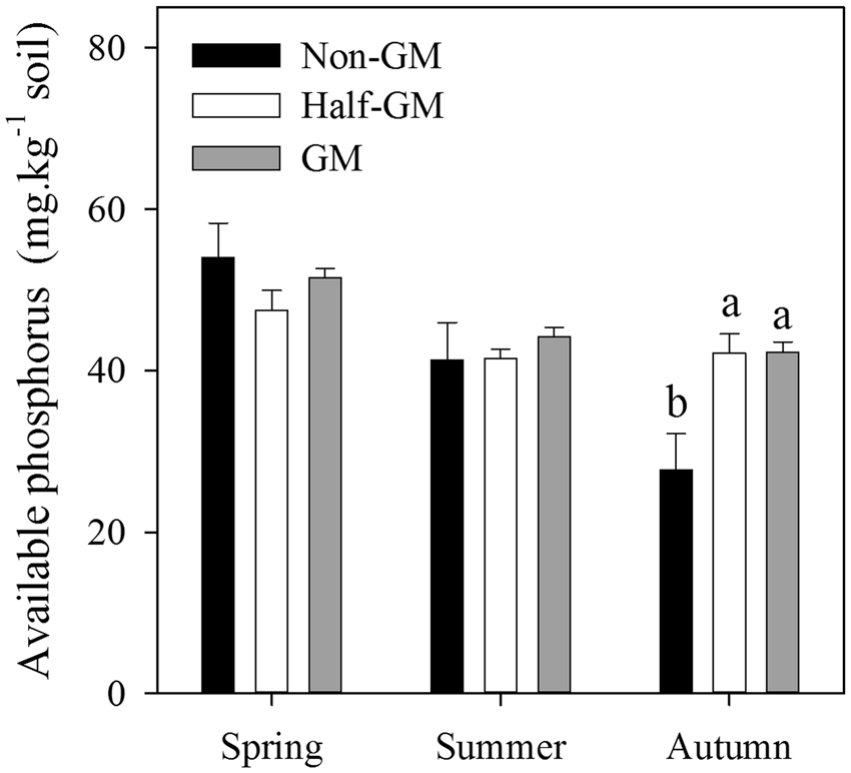
Available phosphorus concentration of *Bt* treatments in different seasons. Error bars represent standard error (n = 4). The same letters over error bars denote no significant difference between treatments (*p* > 0.05).

In spring before rice planting, soils had the highest phosphorous availability, with no significant differences among *Bt* treatments (Fig. 6). In general, soil phosphorus availability among three treatments was similar during summer and autumn. However, after rice harvest, available phosphorus concentration in *Non*-*GM* was significantly lower than those in *Half*-*GM* and *GM* (*p* = 0.016).

**Figure 7:**
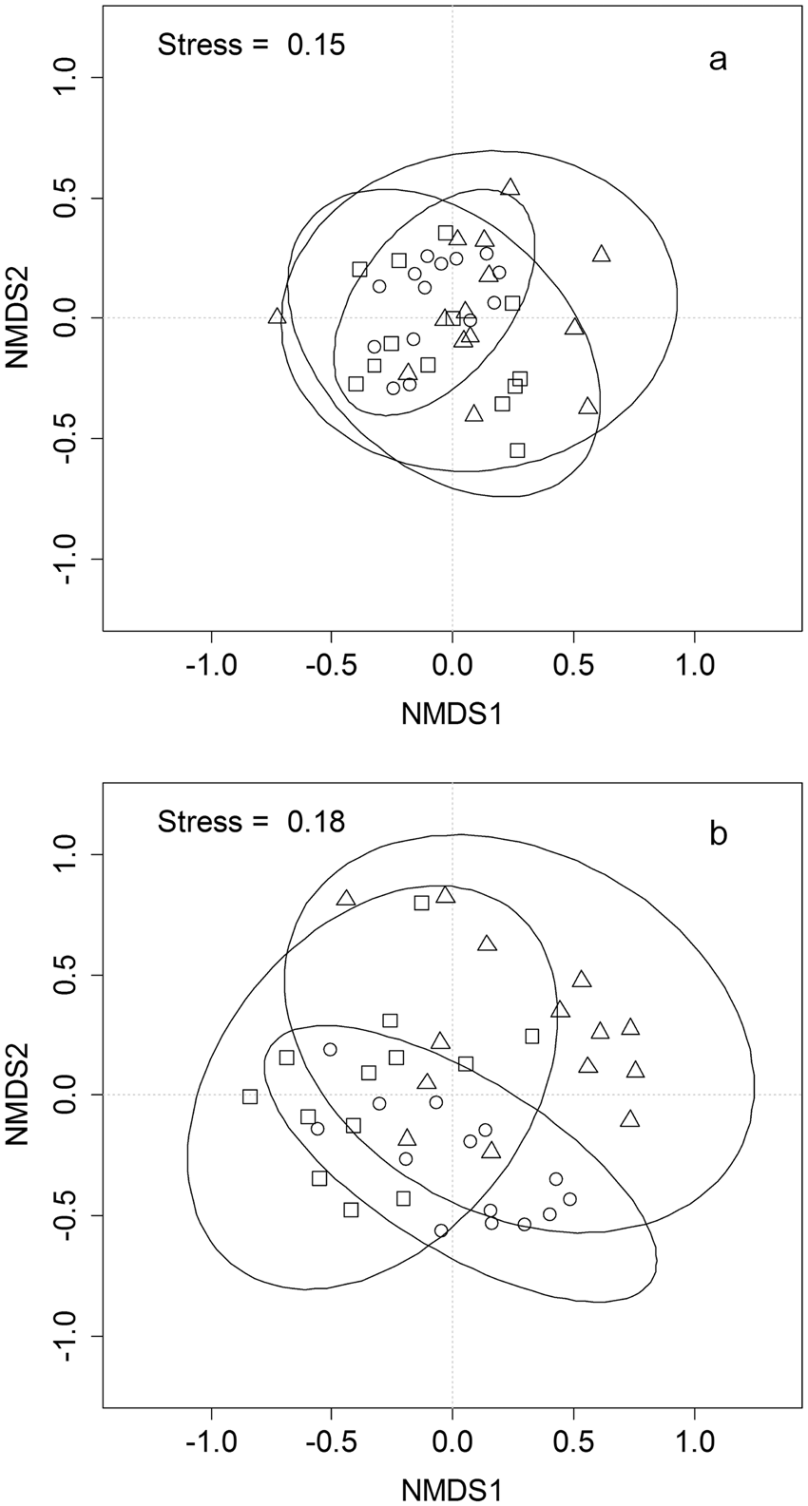
Nonmetric multidimensional scaling (NMDS) analyses of similarities among *Bt* treatments. (**a**) year-round carbon and nutrient pools (TOC, TN, available phosphorus); (**b**) soil enzymatic activities and microbial biomass (catalase, dehydrogenase, acid phosphatase, urease, MBC and MBN). Circles stand for soil samples from *Non-GM*, triangles for *Half-GM* and squares for *GM*. Ellipses were the 95% confidence limits of multivariate means.

The NMDS ordinations, based on year-round C and nutrient pools (Fig. 7a), and soil enzymatic activities and microbial biomass (Fig. 7b), show close overlaps among soils from different treatments, indicating that *Bt* rice does not bring about obvious changes on soil properties throughout the year.

## Discussion

### *Bt* protein in the soil

In general, *Bt* protein concentration in the soil under transgenic *Bt* rice is relatively low, often lower than the minimal detecting level of ELISA method^35^. Higher levels of *Bt* protein concentration, 0.82–2.13 ng g^−1^ dry soil in rhizosphere soils, were reported under cultivation of *Bt*-*Minghui 63* and *Bt*-*Shanyou 63* ^36^. In this study, no *Bt* protein was detected from the soil under *Bt* rice. Based on the amount of root biomass at the time of harvest and *Bt* protein concentration of roots, the annual input of *Bt* protein into the soil through root turnover was estimated at about 1.0 ng g^−1^ in 0–10 cm and 0.24 ng g^−1^ in 10–20 cm depth. The input of *Bt* protein through exudation from live root was difficult to be estimated. *Bt* protein from either root turnover or root exudation should concentrate in rhizosphere rather than in bulk soil. The low concentration of *Bt* protein may render us unable to detect out *Bt* protein in root-free soil samples using ELISA in our study. Another possible reason might be partly related to the short residence time of *Bt* protein in soil. Feng *et al*. pointed out that the half-life of *Bt* protein in the soil varied from 0.75 to 10.89 days, and only 0.02–1.51% remained in the soil after 180 days^37^. Commonly, *Bt* protein cannot be detected in soils at the time of next growing season starting^38^. Our study suggested that long-term cultivation of *Bt* rice is unlikely to result in the accumulation of *Bt* protein in the soil.

### Effects of *Bt* rice on C cycling

The major input of organic matter into the soil ecosystem is from plants through above-ground litterfall, root turnover and root exudation. Changes in crop traits and the quantity and/or quality of C input into the soil may affect soil carbon cycling^39, 40^. It has been reported that *Bt* rice had a different tiller number^41^ and net photosynthetic rate^42^, leading to differences in crop biomass between *Bt* and non-*Bt* crops. Saxena and Stotzky^43^ pointed out that lignin concentration of *Bt* maize was 33–97% higher than those of non-*Bt* ones, partly due to a greater diameter of vascular bundle and surrounding lignin cell. *Bt* gene may also change organic carbon and nitrogen concentrations in *Bt* crops relative to non-*Bt* crops^44, 45^. By contrast, *Bt* rice (*Kefeng-6*) did not have significantly different root biomass, root distributions between surface and sub-surface soil layers, or root C:N ratio compared with its non-*Bt* parental breed (Fig. 1). Although the aboveground biomass of *Bt* rice was significantly higher, it did not result in a higher C input to the soil as most part of aboveground straw was removed or burned after harvest. For a long term and large scale commercial release of *Bt* rice, increased aboveground biomass may lead to a greater amount of C accompanied with *Bt* protein input to the soil.

*In situ* determination of root exudations is still a challenge to ecological research^46^. It was estimated that about 30–60% of photosynthetically fixed carbon was allocated into belowground^47–49^, which then turned into root biomass increment, root respiration and exudation. Under relatively stable environmental conditions, root exudation may be positively correlated with root biomass^50^. Recently, it was reported that root exudation was sensitive to the level of available phosphorus in the soil^51, 52^. Our experimental results of similar root biomass and soil phosphorus availability among *Bt* treatments (Figs 1a and 6) may suggest no significant change in root exudation between *Bt* and non-*Bt* rice.

The reference respiration (*e*.*g*. *R*_20_ in this study) of root-free soil samples defined soil organic matter decomposition rate under given conditions and *Q*_10_ value defined the dependence of TOC decomposition on temperature. As soil water content was kept at 60% of field capacity during soil incubation, *R*_20_ and *Q*_10_ reflected the essential characteristics of TOC decomposition in soils of different *Bt* treatmetns. Meanwhile, due to similar TOC contents in different soil samples, *R*_20_ could also indicate the relative TOC decomposibility, *i*.*e*. the quality of TOC. Our results suggested that after 8 years cultivation of transgenic *Bt* rice *Kefeng-6*, TOC decomposition has not been significantly changed (Fig. 3). *In situ* soil respiration under *Bt* crops has been observed increased^27, 53^ or decreased^25^. Fang *et al*. reported that *Bt* rice (*Huachi-B6*) could significantly reduce anaerobic soil respiration by 22.4% at the heading stage but only 2.78% at filling stage^29^. Field measured soil respiration includes both root respiration and TOC decomposition, regulated by many other factors other than *Bt* protein. The possible mechanisms underlying altered *in situ* soil respiration under *Bt* crops are complicated and still unclear. Changed crop debris input might be one of the reasons that changed soil respiration under *Bt* maize^54^.

### Enzymatic activities and microbial biomass

*Bt* rice did not induce consistently significant changes in soil enzymatic activities after 8-year cultivation, although significant differences among treatments were observed in summer time when *Half*-*GM* and *GM* treatments reduced catalase activities and *GM* increased dehydrogenase and acid phosphatase activities (Fig. 2). These results from *Bt* rice seemed to be different from previous studies on other *Bt* crops. It was reported that soil enzymatic activities including dehydrogenase and acid phosphatase were lowered after *Bt* cotton consecutively planted for 3–5 years^55^. Sarkar *et al*. found that *Bt* cotton issued in higher enzymatic activities, MBC, MBN, and MBC/TOC^21^. There may be two possible reasons, *i*.*e*. *Bt* protein concentration and the amount of crop debris input to soil ecosystem, accounting for this divergence. *Bt* concentrations under *Bt* rice cultivations were much lower than other *Bt* crops where *Bt* protein concentration could be up to 56.14 ng g^−1^ under *Bt* cotton^22^. Besides, due to aboveground litter removed, there were no significant differences in crop debris input to soil between *Bt* rice and non-*Bt* rice in this study. Our results were consistent with reported short-term studies on *Bt* rice^19^. Eight years cultivation of *Bt* rice (*Kefeng-6*) has not significantly nor consistently changed soil enzymatic activities. The results of MBC and MBN in this study revealed that there were no consistently significant differences among treatments after 8 years cultivation (Fig. 4). Similar results were observed in other short-term studies^21, 30^ or long-term studies on *Bt* maize^36, 56^. The fact that microbial metabolic quotients among *Bt* rice treatments were close to one another also suggested that soil microbial biomass was not affected by *Bt* rice *Kefeng-6*.

### Effects of long-term *Bt* rice cultivation on soil ecosystem

Agricultural soil is a complex system under intensive human disturbances. Some components of the system, such as enzymatic or microbial activities, respond instantaneously and sensitively to environmental changes. In most cases, these responses are reversible if changed environmental factors return to previous situation. Furthermore, sensitive components of soil system are often highly heterogeneous and variable^57, 58^. The other components of soil ecosystem are relatively insensitive to environmental changes, such as TOC. The amount of TOC reflects an accumulative result of changed influencing factors over a relatively long period. The amount of TOC may stay stable even if changed influencing factors return to previous stituation. So far, the effects of transgenic *Bt* crops on soil ecosystem have been assessed seperately on enzymatic activities^19, 29^, TOC^21, 40^, soil respiration^56^ and microbial biomass^21, 26^ in a short term and significant differences were occasionally observed. However, taking into consideration all these arguments in this study, no consistent changes have been observed in soil enzymatic activities, microbial biomass, C cycling after 8 years cultivation of transgenic *Bt* rice *Kefeng-6*, suggested that soil ecosystem had not been irreversibly changed by *Bt* rice (Fig. 7). With a large scale commercial release of *Kefeng-6*, increased aboveground biomass may increase organic matter input to the soil, resulting in an increase in TOC and some irreversible changes of soil ecosystem, but these possible changes are unlikely to be negative.

The experimental site of this study was initially constructed for *Bt* rice breeding, a fully random design of soil sampling procedure at this site was practically impossible. The pseudo-replication of sampling procedure may bring in some uncertainties to results due to possibly heterogenous background among different treatments. Indeed, pseudo-replication design cannot statistically factor out the influences of background heterogeneity from measured differences among treatments. However, this pseudo-replication was unlikely to cause a serious bias to measured results and conclusion that there was no significant differences among different *Bt* treatments. In this study, coefficients of variation (CV) of measured soil variables showed no consistent tendences in different seasons, as well as in different treatments (data not shown). Taking the activities of catalase for example, the greatest CV was observed in *Half-GM* (0.15), and *Non-GM* and *GM* was at the similar level (approximately 0.10) before rice planting. Whereas, the greatest value apeared in *GM* and the lowest one in *Non-GM* after rice harvest. In addition, the greatest CV of MBC appeared in *Non-GM* in spring (0.11), *GM* in summer (0.08), and *Half-GM* in autumn (0.06), respectively. No systematical or significant differences in soil variables was observed within and between treatments. This suggested that either the influence of background heterogenity was relatively small, or the agro-ecosystem has a capacity to buffer slight differences in soil background.

## Conclusions

After 8 years cultivation of transgenic *Bt* rice *Kefeng-6*, no *Bt* protein was detected out from the soil. In addition, soil enzymatic activities, microbial biomass and metabolic quotient, soil organic carbon pools and decomposition have not been significantly nor consistently changed. Although the net primary productivity of *Bt* rice was greater than that of non-*Bt* rice, C allocation to belowground were similar. Due to above-ground straw was removed after rice harvest, increased net primary productivity by *Bt* rice has not resulted in significant changes in soil C cycling. From the aspects of soil microbial functioning and C cycling, long-term cultivation of transgenic *Bt* rice *Kefeng-6* is unlikely to cause irreversibly effects on soil ecosystem.

## Materials and Methods

### Site and experimental treatments

**Figure 8:**
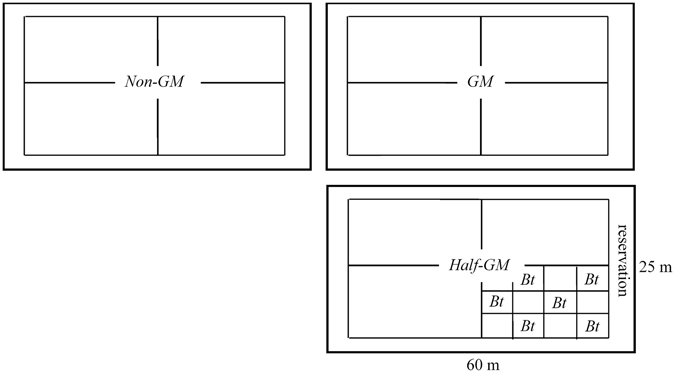
The schematic diagram of experimental design. *Non-GM* treatment was always planted non-*Bt* rice (*Minghui-86*); *GM* was planted *Bt* rice (*Kefeng-6*); and *Half-GM* was a treatment where *Bt* rice was planted in one plot and non-*Bt* rice was planted in all adjacent plots. In consecutive year, *Bt* plot was changed to non-*Bt* plot, and vice versa. Reservation area was always planted non-*Bt* rice.

Field experiment was estabished in 2002 in an authorized and confined Biosafety Experimental Field at Wufeng Village of Fuzhou City, Southeastern China. Three experimental blocks (Fig. 8) were set up for: (1) *GM* treatment, continuously growing transgenic *Bt* rice (*Kefeng-6*, encoding *Cry1Ac* protein with *lepidopteron* being the target insect); (2) *Half*-*GM* treatment, transgenic *Bt* rice (*Kefeng-6*) and non-transgenic rice (*Minghui-86*, the parent breed of *Kefeng-6*) being alternatively cultivated in subsequent years, and (3) *Non*-*GM* control, continuously growing non-transgenic rice *Minghui-86*. The dimension of each block was about 60 × 25 m. Each block was separated from others by a hard dam and had an independent irrigation system. *Half*-*GM* block was divided into 3 × 3 m plots. *Bt* rice (*Kefeng-6*) and non-*Bt* rice (*Minghui-86*) were planted in adjacent plots. In the consecutive year, *Bt* plots were changed to non-*Bt* plots, and vice versa. Each block was split into four sub-blocks, which could be considered as pseudo-replications. All treatments were under traditional management.

### Soil and plant roots sampling

Soil samples were collected from 0–20 cm depth in spring (before rice planting), summer (rice growing season), and autumn (after rice harvest). A soil auger of 5 cm inside diameter was used to collect soil. For *GM* and *Non-GM* treatments, 5 locations were randomly selected in each sub-block. Collected soils from these 5 locations were composited into a soil sample. For *Half-GM* treatment, one location was randomly selected in each plot, and collected soils were then composited to obtain one soil sample. After visible roots removed, all soil samples were transported to laboratory within 24 h. A subsample was immediately kept at 4 °C for enzymatic activity analyses (except for catalase) and the remaining part was milled to 2 mm and also kept at 4 °C for chemical, biological analyses and soil incubation. For each soil sample, a sub-sample of roughly 10 g was air-dried and milled to 0.15 mm for TOC and TN measurements. Soil properties of the three treatments are presented in Table 1.

**Table 1:** Some basic soil (0–20 cm) properties.

	pH	Bulk density (g.cm^−3^)	Sand (%)	Silt (%)	Clay (%)
*Non-GM*	5.20 (0.08)	2.43 (0.04)	11.40 (1.14)	50.80 (1.14)	37.8 (0.45)
*Half-GM*	5.30 (0.04)	2.21 (0.04)	24.65 (0.95)	41.55 (0.95)	33.3 (0.00)
*GM*	5.07 (0.02)	2.25 (0.04)	15.65 (0.85)	47.55 (0.85)	36.8 (0.00)

Notes: standard error is shown in parentheses.

Aboveground rice biomass and roots were collected just before rice harvest. For aboveground biomass sampling, five quadrats of 50 × 50 cm^2^ were randomly selected in each treatment site. Aboveground biomass was clipped to ground level and separated into stem, leaf and seed. Surface litter was then carefully removed. A soil cube of 25 × 25 × 10 cm was taken from 0–10 and 10–20 cm depths in each quadrat. Soil cubes were washed manually over a 2-mm mesh size nylon net using clear water. All plant samples were then washed with deionized water and dried at 60 °C to constant weight. Subsamples of root, stem and leaf were milled to 0.15 mm for measurement of TOC and TN. These analyses were conducted within 6 months after samplings.

### Chemical and biological analyses

Soil pH was determined at a soil: water ratio of 1:5 (w/v). Available soil phosphorus content was colorimetrically determined^59^. TOC and TN were analyzed on a NC soil analyzer (Flash EA 1112 series; Thermo Finnigan, Elk Grove Village, IL), with combustion at 625 °C and 900 °C, respectively. *Bt* protein concentration in the soil was determined using ELISA approach with the phosphate-buffered saline Tween 20 and the detailed procedures was in accord with Xiao *et al*.^60^, which has a generally minimal detecting level of 0.5 ng g^−1^ dry soil^35^.

Soil MBC and MBN were analyzed using the chloroform fumigation-extraction method^61^. After pre-incubated at 25 °C for 15 days, two fumigated and two non-fumigated soil samples, 20 g dry weight equivalent, were extracted with 80 ml 0.5 M K_2_SO_4_ and horizontally shaken at 250 rev min^−1^ for 45 min. Carbon and nitrogen concentrations in the extracts were analyzed with a TOC analyzer (Multi N/C 3100, Jena, Germany). MBC and MBN were estimated following Wu *et al*.^61^, taking *K*_*EC*_ = 0.45.

### Enzymatic activities

Catalase was determined by titration with KMnO_4_^62^. Forty milliliter deionized water and 5.0 ml 0.3% hydrogen peroxide solution were added to 2 g air-dried soil sample (milled to 1 mm). The mixture was shaken at 150 rev min^−1^ for 20 min, followed by adding 5.0 ml H_2_SO_4_ (1.5 mol L^−1^) to end the reaction. The solution was filtered and titrated using 0.02 mol L^−1^ KMnO_4_. Catalase activity was finally expressed as 20 min KMnO_4_ 0.02 mol L^−1^.

Acid phosphatase activity was determined using P-nitrophenyl phosphate disodium as substrates^63^. One gram moist soil was put into a 50 ml centrifugal tube, and then 0.25 ml toluene, 4.00 ml MUB buffer (pH 6.5) and 15 mmol L^−1^ P-nitrophenyl phosphate disodium were added. The mixture was incubated at 37 °C for 1 hour. Chemical reaction was ended by adding 4.00 ml CaCl_2_ (0.5 mol L^−1^) and 4.00 ml NaOH (0.5 mol L^−1^). The soil suspension was filtered and the resulting solution was measured at 400 nm using a UV-VIS spectrometer (T6, Beijing Purkingje General Instrument, China). The activity of acid phosphatase was expressed as *μ*g PNP g^−1^ dry soil h^−1^.

Dehydrogenase activity was measured by the reduction of triphenyl tetrazolium chloride (TTC) to 2, 3, 5-triphenyl formazan (TPF)^64^. Ten gram moist soil was throughly mixed with 0.1 g CaCO_2_, and then 3 g mixture was dispensed to a 50 ml centrifugal tube. Soil sample was added with 0.5 ml 3% TTC and 1.25 ml deionized water, and incubated at 37 °C for 24 hours. Reaction was ended by adding 5.0 ml toluene. Mixture in the tube was centrifuged at 4000 rev min^−1^ for 5 min to extract TPF. The extraction was then added to 50.0 ml using toluene. The optical density of extracted supernatant was measured at 485 nm on a UV-VIS spectrometer (T6, Beijing Purkingje General Instrument, China). Soil dehydrogenase activity was expressed as *μ*g TPF g^−1^ dry soil.

To analyze urease activity, approximate 5 g soil was placed into a 50 ml volumetric flask mixed with 1 ml phenol. Fifteen minutes later, 5 ml urea solution (100 g urea L^−1^) and 5 ml citrate solution (pH 6.7) were added to soil sample which was then incubated at 38 °C for 3 hours. The mixture was then diluted to 50 ml using deionized water (38 °C). The suspension was filtered, and a aliquot (1.0 ml) filtered solution was moved into a 50 ml volumetric flask and diluted to 10 ml with deionized water; 4 ml sodium phenol and 3 ml sodium hypochlorite were then added and mixed; 20 min later, the mixture was diluted to 50 ml with deionized water. The final solution was analyzed on a spectrophotometer (T6, Beijing Purkingje General Instrument, China) at 578 nm within 1 hour. Urease activity was presented as mg NH_4_^+^ -N g^−1^ dry soil h^−1^ ^65, 66^.

All soil samples were conducted with 3 replicates and enzymatic activities were represented as the average of replicates.

### Soil incubation and respiration measurement

Soil incubations followed Chen *et al*.^67^. Fresh soil samples, equivalent to 30 g dry weight, were incubated in 300 ml jars under changing temperature. Soil moisture was adjusted to and maintained at 60% of field holding capacity during the whole incubation by adding deionized water every two days to recover weight loss^67, 68^. All samples were pre-incubated at 25 °C for 48 hours to minimize initial disturbances. Fresh air was continuously passed through the head space of incubation jar at a flow rate of 0.75 L min^−1^. Incubation temperature was increased from 10 °C to 31 °C with a step length of 3 °C and then backed to the minimum^69^. Soils were kept at each temperature for 4–10 hours, of which the first 2 hours were allowed for achieving a new equilibrium after temperature changed and the following hours for respiration measurement.

Five milliliter gas was sampled from the head space immediately after incubation jars were closed. Whereafter, a same volume of CO_2_-free air was immediately injected into jars to balance pressure. After a certain period, depending on CO_2_ concentration change in the head space, a second gas sample of 5 ml was taken and incubation jar was then opened to allow fresh air flowing through. The actual volume of head space was measured by water replacement at the end of incubation. Soil respiration rate was calculated as CO_2_ concentration differences between the first and the second gas samples, based on the volume of head space and the period of jar closure.

To measure the temperature sensitivity of soil C mineralization, soil respiration rates were fitted to temperature by an exponential function as below:

1 $$R=a{e}^{bt}$$

where *R* is soil respiration rate in *μ*mol g^−1^ dry soil h^−1^, *t* is temperature (°C), *a* and *b* are fitting parameters, respectively. A reference soil respiration rate, *R*_20_, was defined as the respiration rate at 20 °C.

The temperature sensitivity of soil respiration, measured by *Q*_10_ value, was calculated by:

2 $${Q}_{10}={e}^{10b}$$

Soil microbial metabolic quotient, *q*CO_2_, was defined as:

3 $$qC{O}_{2}={R}_{20}/MBC$$

### Data analysis

All data were analyzed using a software of SPSS for Windows, 21.0 version. The *t*-test were used to compare biomass between *Bt* rice and non-*Bt* rice. The other data were analyzed by one-way factorial analysis of variance. Post hoc pairwise multiple comparisons were conducted using the Duncan tests. To test whether there were changes in each type of soil enzymatic activity, carbon and nutrient pools, a nonmetric multidimensional scaling (NMDS) based on Bray-Curtis distances was performed to graphically visualize the organization of samples in two-dimensional space, using a *R* multivariate statistical analysis package: Vegan^70^.
